# The Metabolic Landscape of Cancer Stem Cells: Insights and Implications for Therapy

**DOI:** 10.3390/cells14100717

**Published:** 2025-05-15

**Authors:** Martina Milella, Monica Rutigliano, Savio Domenico Pandolfo, Achille Aveta, Felice Crocetto, Matteo Ferro, Antonio d’Amati, Pasquale Ditonno, Giuseppe Lucarelli, Francesco Lasorsa

**Affiliations:** 1 Urology and Kidney Transplantation Unit, Department of Precision and Regenerative Medicine and Ionian Area-Urology, University of Bari “Aldo Moro”, 70124 Bari, Italy; martina.milella97@gmail.com (M.M.); antonio.damati@uniba.it (A.d.);; 2 Department of Urology, University of L’Aquila, 67100 L’Aquila, Italy; pandolfosavio@gmail.com (S.D.P.); felice.crocetto@unina.it (F.C.); 3 Department of Neurosciences, Science of Reproduction and Odontostomatology, Federico II University, 80138 Naples, Italy; 4 Urology Unit, Department of Health Science, University of Milan, 20122 Milan, Italy; 5 SSD Urologia Clinicizzata, IRCCS Istituto Tumori “Giovanni Paolo II”, 70124 Bari, Italy

**Keywords:** cancer, stem cells, markers, treatment

## Abstract

Cancer stem cells (CSCs) are a subpopulation with self-renewal and differentiation capacities believed to be responsible for tumor initiation, progression, and recurrence. These cells exhibit unique metabolic features that contribute to their stemness and survival in hostile tumor microenvironments. Like non-stem cancer cells, CSCs primarily rely on glycolysis for ATP production, akin to the Warburg effect. However, CSCs also show increased dependence on alternative metabolic pathways, such as oxidative phosphorylation (OXPHOS) and fatty acid metabolism, which provide necessary energy and building blocks for self-renewal and therapy resistance. The metabolic plasticity of CSCs enables them to adapt to fluctuating nutrient availability and hypoxic conditions within the tumor. Recent studies highlight the importance of these metabolic shifts in maintaining the CSC phenotype and promoting cancer progression. The CSC model suggests that a small, metabolically adaptable subpopulation drives tumor growth and therapy resistance. CSCs can switch between glycolysis and mitochondrial metabolism, enhancing their survival under stress and dormant states. Targeting CSC metabolism offers a promising therapeutic strategy; however, their adaptability complicates eradication. A multi-targeted approach addressing various metabolic pathways is essential for effective CSC elimination, underscoring the need for further research into specific CSC markers and mechanisms that distinguish their metabolism from normal stem cells for successful therapeutic intervention.

## 1. Introduction

Cancer stem cells (CSCs) are a subpopulation of cells with the ability to self-renew and differentiate and are thought to be responsible for tumor initiation, progression, and recurrence. These cells exhibit unique metabolic characteristics that contribute to their stemness and survival in harsh tumor microenvironments [1]. The metabolism of CSCs is distinct from that of non-stem cancer cells, as well as normal tissue cells.

Several studies have shown that CSCs heavily rely on glycolysis for energy production, similar to the Warburg effect observed in non-stem cancer cells. This metabolic shift toward glycolysis allows them to efficiently generate adenosine triphosphate (ATP) and sustain their rapid proliferation. In addition to glycolysis, CSCs also exhibit an increased reliance on other metabolic pathways, such as oxidative phosphorylation (OXPHOS) and fatty acid metabolism. These alternative metabolic pathways provide them with the necessary building blocks and energy sources to support their self-renewal and resistance to therapies [2]. The metabolic plasticity of these cells allows them to adapt to changing nutrient availability and hypoxic conditions in the tumor microenvironment.

In this review, we describe recent evidence about CSC metabolism and its role in cancer progression.

## 2. CSC Characteristics

While normal stem cells assist in the differentiation of normal tissues, cancer tissues are made up of a hierarchical structure of differentiated cells derived from cancer stem cells [3,4,5,6]. Cancer is defined as the uncontrolled proliferation of abnormal cells with varying structures and functions [7,8,9,10,11]. Two primary hypotheses have been proposed to explain the cellular diversity found in tumors. The first is the classic stochastic model, which describes cancer genesis and development as the gradual accumulation of mutations. The second approach, known as the cancer stem cell hypothesis, suggests that tumor growth is driven by a limited population of stem cells [12,13]. The presence of cancer stem cells may play a significant role in treatment resistance and recurrence. Recent research has shown that CSCs undergo metabolic changes that set them apart from non-CSCs, raising the possibility of developing curative therapies for CSCs by targeting specific metabolic pathways. However, there is debate over whether CSCs rely more on glycolysis or mitochondrial OXPHOS to maintain their stem cell characteristics [14,15,16]. CSCs have a notable migratory potential, allowing them to spread from the primary tumor to distant organs [17,18]. Various methods have been developed to isolate and characterize CSCs. They are typically found in specialized niches where they depend on specific growth factors like epidermal growth factor (EGF) and fibroblast growth factor (bFGF) to maintain their undifferentiated state and support self-renewal [19,20]. In addition to their ability to self-renew in an undifferentiated state, CSCs share many common features with normal stem cells, including the expression of surface markers (CD44, CD133, aldehyde dehydrogenase), the activation of specific signaling pathways (Notch, Hedgehog, or Wnt, relative quiescence), and the capacity to actively repair DNA [8,18,19,20,21,22].

Depending on the type of cancer, CSCs can be identified by a variety of surface markers, such as CXCR4, ESA, and Nestin. For example, in pancreatic cancer, CSCs are identified by combinations like CD133+/CXCR4+, CD24+/CD44+, and other profiles, while in breast cancer, they express CD24−/low/CD44+ [23,24]. In glioblastoma, CSCs are characterized by CD133+/ABCG2+ [25,26]. CD44 appears to play a role in tumor metastasis and invasion by facilitating CSC attachment to the extracellular matrix, promoting tumor cell migration, and contributing to metastatic spread by binding to its ligand hyaluronic acid [27,28,29,30].

Previous investigations have emphasized the role of renal stem/progenitor cells in kidney regeneration following tubular damage. These cells, characterized by markers like CD133 and CD24, possess self-renewal capabilities and the potential to differentiate into various renal cell types, such as tubular cells and podocytes. While CD133+ progenitor cells isolated from renal tumors are not tumorigenic by themselves, they can enhance tumor development and angiogenesis when co-transplanted with malignant cells, indicating their contribution to cancer progression. Further research identified a subset of CD105+, CD133+, and CD24+ CSCs with mesenchymal properties, which exhibit resistance to chemotherapy and demonstrate self-renewal and multipotency. Markers such as CD44 and CXCR4 are also vital for RCC CSCs [31]. CD44 is involved in cell migration, adhesion, and signaling, activating pathways like TGFβ, MAPK, and PI3K/AKT that support tumorigenesis. Meanwhile, CXCR4 directs CSCs to metastatic sites by interacting with SDF1, promoting cancer spread. These CSCs are highly resistant to chemotherapy, mainly due to the activation of the Notch and Wnt/β-catenin pathways, contributing to survival and drug resistance. Furthermore, CTR2, a copper transporter, influences drug resistance by modulating the uptake of platinum-based chemotherapies [19]. This transporter regulates copper balance in the cell, which can impact the effectiveness of treatment. RCC CSCs are pivotal in tumor growth, metastasis, and chemoresistance, highlighting their potential as therapeutic targets.

## 3. Glucose Metabolism in CSCs

**Figure 1:**
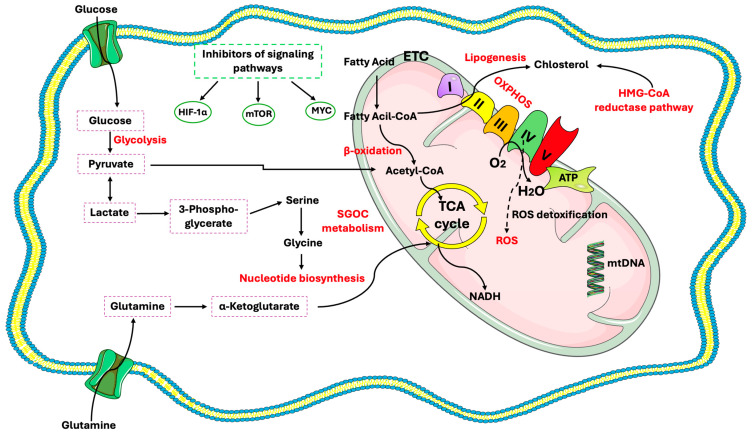
Metabolic features of CSCs and related therapeutic targets. CSCs exhibit high metabolic flexibility, alternating between glycolysis and OXPHOS to support stemness and adapt to stress. Key pathways, including serine/glycine one-carbon metabolism, glutaminolysis, lipid metabolism, and the TCA cycle, are regulated by oncogenes like MYC, mTOR, and HIF-1α. Targeting these metabolic nodes offers promising strategies to eliminate CSCs and prevent tumor recurrence.

Glucose is a primary energy source for both CSCs and differentiated tumor cells, offering rapid energy but with lower efficiency (Figure 1). Under normal oxygen conditions, pyruvate from glycolysis is used in OXPHOS to produce ATP [32]. In an anaerobic environment, pyruvate is converted to lactate by lactate dehydrogenase-A (LDH-A) and exported via monocarboxylate transporters (MCT) [33]. While glycolysis provides rapid ATP, especially under hypoxic conditions, its energetic yield is much lower than OXPHOS. OXPHOS remains a key pathway for energy production necessary for tumor growth, even in tumor cells [34,35,36,37,38]. Apart from ATP production, pyruvate and other glycolytic intermediates are crucial for amino acid and lipid biosynthesis (via the tricarboxylic acid cycle, TCA, and acetyl-CoA production, respectively). This is essential for the rapid proliferation of metastatic cancer cells, which require both energy and biosynthetic precursors within a short time frame [39,40,41]. Elevated glucose levels and increased expression of glucose transporters promote glycolysis and are associated with enhanced tumor cell and CSC viability. Glucose transporters can be classified into two types according to their mechanism of action: GLUT transporters and sodium-dependent SGLT transporters, which operate against the gradient by coupling with sodium ions [42,43,44]. In CSCs, GLUTs and SGLTs play distinct but complementary roles in glucose uptake. GLUTs, particularly GLUT1, are primarily responsible for the passive transport of glucose into the cell, supporting the high glycolytic activity and metabolic demands of CSCs, especially in low-oxygen environments. They help maintain CSC properties like self-renewal and resistance to therapies. On the other hand, SGLTs actively transport glucose along with sodium ions, which is particularly useful when glucose levels are low outside the cell. This process ensures that CSCs can still take up glucose under nutrient-poor conditions, supporting their survival [36]. CSCs exhibit increased glucose uptake due to their reliance on high glycolytic activity and low OXPHOS [45]. Reducing glucose concentration or inhibiting GLUT1 via genetic knockdown or pharmacological agents limits stemness and spheroid formation in pancreatic, ovarian, and glioblastoma CSCs without compromising cell viability [46]. Even so, CSCs are more glycolytic than differentiated cancer cells in a variety of malignancies [47,48,49]. Upregulation of glycolytic genes occurs before the expression of pluripotency markers, indicating that switching from OXPHOS to glycolysis promotes stemness in CSCs rather than pluripotency [50]. CSCs have greater glucose absorption and, hence, lactate and ATP generation, and die when glycolysis is inhibited or glucose is depleted [51,52]. Glycolytic CSCs are observed in CD133+ osteosarcoma-initiating cells, glioblastoma cells, breast cells, and liver cancer cells [33,47,48,49,50,51,52,53,54,55].

Lactate plays a key role in maintaining stem cell characteristics by upregulating the transcription factor SP1 (Specificity Protein 1), which, in turn, enhances tumor aggressiveness, invasiveness, and immune evasion via sterol regulatory element-binding protein 1 (SREBP1) [56,57,58,59,60,61]. In CSCs, hypoxia-inducible factor-1 (HIF-1) shifts metabolism toward glycolysis while suppressing OXPHOS and the tricarboxylic acid cycle [62]. HIF-1 also reduces reactive oxygen species (ROS) production and induces the expression of GLUT, hexokinase 2, pyruvate kinase, and lactate dehydrogenase A while downregulating pyruvate dehydrogenase [52]. These metabolic adaptations support CSC self-renewal and pluripotency, contributing to resistance to therapies across various types of cancer [63,64]. Consequently, it can be proposed that the metabolic profiles of cancer stem cells may exhibit variability, influenced by both the tissue of origin and the specific metastatic niche.

### Mitochondria in CSCs

Beyond serving as a primary ATP source for cancer cells, mitochondria are involved in regulating various signaling pathways. These include the release of cytochrome C to trigger apoptosis, ROS generation, and the synthesis of metabolites like acetyl-CoA, which plays a role in modulating protein acetylation [11,65,66,67]. Mitochondria are responsible for producing over 90% of cellular ATP via OXPHOS [68]. In contrast, mitochondrial dysfunction leads to the suppression of OXPHOS in most CSCs. When OXPHOS is upregulated, it is closely linked to processes like epithelial–mesenchymal transition (EMT) and tumor metastasis [69]. In environments with limited nutrients, oxidative stress stimulates tumor cells to rely on glycolysis for lactate and metabolite production, while OXPHOS continues to be the dominant pathway for cellular metabolism, fueling anabolic processes that drive tumor progression [70].

Recent investigations have demonstrated that CSCs from various tumor types, such as ovarian, cervical, lung, and glioma, exhibit an enhanced reliance on mitochondrial function and OXPHOS compared to their non-stem counterparts [20]. For instance, ovarian cancer-derived CSCs show increased expression of enzymes involved in OXPHOS and fatty acid metabolism [71]. Likewise, spheroids from ovarian and cervical carcinomas display a reprogrammed metabolic state, favoring the TCA cycle relative to non-CSCs [72]. In small-cell lung cancer, CSCs are more dependent on OXPHOS and mitochondrial activity than their non-stem cell counterparts [73]. Glioma-derived CSCs utilize less glucose and lactate while sustaining high ATP production via OXPHOS [74]. This pattern is also seen in CD133+ glioblastoma cells, where the overexpression of insulin-like growth factor 2 mRNA-binding protein 2 (IMP2) modulates mitochondrial dynamics and stemness markers such as CD133, SOX2, and NANOG [75]. Moreover, dormant cells surviving RAS ablation in pancreatic cancer show a similar reliance on mitochondrial function and OXPHOS instead of glycolysis to preserve stem cell properties [76]. A strong association between the metastatic potential of cancer cells and the transcriptional co-activator PGC-1α (PPARGC1A) has been reported [77]. PGC-1α has been found to play a pivotal role in regulating mitochondrial functions, including OXPHOS and mitochondrial biogenesis, which, in turn, contributes to enhanced cellular migration and invasion, as demonstrated in invasive breast cancer models. Additionally, PGC-1α overexpression has been observed in circulating tumor cells and breast CSCs, with suppression of its activity leading to reduced stem cell characteristics [78]. These findings highlight the essential role of mitochondrial integrity in sustaining CSC properties. Consequently, mitochondrial biogenesis has become recognized as a central feature of CSCs, which exhibit higher mitochondrial mass, increased membrane potential, augmented mitochondrial ROS production, and elevated oxygen consumption relative to differentiated tumor cells [79,80,81,82,83].

Consistent with these findings, mitochondrial activity and metabolic processes could play a key role in the spread of CSCs. Since preserving a functional mitochondrial network is critical for sustaining and propagating stem cell characteristics, targeting these organelles may offer a promising therapeutic approach to eliminate CSCs.

## 4. Lipid Metabolism in CSCs

Rapidly proliferating cancer cells have an increased requirement for lipids and cholesterol [84,85,86,87]. This need is fulfilled through enhanced uptake of external lipids and lipoproteins or, alternatively, by the activation of intracellular metabolic pathways that drive the synthesis of lipids and cholesterol, such as lipogenesis and the mevalonate pathway [88,89,90,91]. In this context, the lipid metabolism of CSCs is of particular interest. In cell membranes, lipids, particularly cholesterol and sphingolipids, form microdomains known as lipid rafts, which in cancer cells contain various signaling molecules and receptors involved in both oncogenic and apoptotic pathways [92]. Within the stem cell compartment, both hematopoietic stem cells and leukemia-initiating cells are highly reliant on fatty acid oxidation for their energy needs [93,94]. Interestingly, leukemia-initiating cells take advantage of the adipose tissue microenvironment to create conditions that support leukemic growth and resistance to chemotherapy. They promote lipolysis in gonadal fat, which leads to the release of free fatty acids that fuel fatty acid oxidation in leukemia-initiating cells, a process facilitated by upregulation of the fatty acid transporter CD36. A similar role for CD36 has also been observed in oral CSCs [95].

Lipid accumulation occurs in cancer cells in lipid droplets, likely originating from the endoplasmic reticulum or Golgi apparatus. Increased droplet levels are linked to tumor aggressiveness, and they have been detected in circulating tumor cells, with their quantification suggesting a prognostic tool for survival outcomes [96,97,98]. An elevated amount of droplets is a characteristic feature of CD133+ colorectal (CR)-CSCs, as demonstrated by Raman spectroscopy [99].

Unsaturated lipids are critical for the function and progression of various types of tumors, including breast, colon, renal, and prostate cancer [100,101]. Administering unsaturated fatty acids into mice with pre-established colorectal cancer increases specific subpopulations of CSCs, which enhances their stem-like traits and fosters both tumor growth and metastasis [102]. One key player in this process is stearoyl-CoA desaturase, an enzyme responsible for introducing double bonds into fatty acid chains [103]. This desaturation increases membrane fluidity, a crucial factor for maintaining the integrity and functions of cancer cells. In ovarian cancer, CSCs are particularly reliant on unsaturated lipids, which differentiate them from non-stem tumor cells. Inhibiting lipid desaturases in these cells reduces markers associated with stemness and prevents the initiation of tumors. This suggests that targeting lipid desaturation could serve as an effective strategy for limiting tumor growth and spread [104]. Lipid desaturation also plays a role in modifying membrane characteristics, which are key for cellular processes like division, migration, and signaling critical for the metastatic potential of cancer cells. Moreover, lipid desaturation interacts with several oncogenic pathways, including NF-kB, Wnt, and Hippo/YAP, all of which are involved in CSC regulation. These pathways are enhanced by unsaturated lipids, which promote the stabilization of important proteins like β-catenin and YAP, reinforcing CSC properties and survival [105]. Additionally, unsaturated fatty acids can trigger mesenchymal stem cells to release angiogenic factors such as IL-6, VEGF, and nitric oxide, further facilitating tumor blood vessel formation and metastasis. Lipid desaturation significantly influences the maintenance and function of CSCs, playing a crucial role in tumor progression and metastasis. Targeting lipid desaturases may provide a promising therapeutic approach for inhibiting tumor growth and reducing the risk of cancer recurrence and metastasis.

### 4.1. Key Lipid Regulators

Stearoyl-CoA desaturase 1 (SCD1) is an enzyme that converts saturated fatty acids into monounsaturated fatty acids (MUFAs) [106]. It is highly upregulated in various cancers and is associated with tumor progression and poor clinical outcomes. SCD1 plays a key role in the generation and maintenance of stem cell properties in CSCs and tumor-initiating cells (TICs), particularly in liver, breast, and ovarian cancers [107,108,109,110]. Moreover, SCD1 overexpression promotes CSC proliferation and inhibits apoptosis [111]. The increased activity of SCD1 and subsequent MUFA production are emerging as defining characteristics of CSCs.

Sterol regulatory element-binding protein 1 (SREBP1) is a critical transcription factor involved in the synthesis of fatty acids and cholesterol [112]. It is a central regulator of lipogenesis, influencing the expression of key enzymes like ATP citrate lyase (ACLY), acetyl-CoA carboxylase (ACC1), and fatty acid synthase (FASN) [113]. Overexpression of SREBP1 has been shown to support tumor proliferation and sustain the stem-like properties of CSCs [81]. Additionally, SREBP1 can upregulate the expression of SCD1 [55,93]. This dual role of SREBP1 in regulating lipid metabolism and supporting CSCs highlights its significance in both cancer progression and stem cell biology [114,115].

HMG-CoA reductase (HMG-CoAR) is the rate-limiting enzyme in the mevalonate pathway that is crucial for cholesterol, steroid hormones, and non-sterol isoprenoid synthesis. This enzyme is also the primary target of statins, commonly used to reduce serum cholesterol levels [116,117,118,119]. The pathway generates intermediates like farnesyl pyrophosphate (FPP) and geranylgeranyl pyrophosphate (GGPP), which are critical for protein prenylation, a process that ensures the proper membrane attachment of small GTPases from the Ras and Rho families. Interestingly, inhibition of this pathway by simvastatin has been shown to decrease the number of CSCs, which could have potential implications for future cancer therapy [120,121,122,123].

### 4.2. Lipid Droplets

Lipid droplets (LDs) are cellular organelles that store lipids and are surrounded by a phospholipid monolayer [124,125,126]. In cancer cells, LDs are more abundant compared to normal cells [127]. When glycolysis is inhibited, LD-derived free fatty acids (FFAs) contribute to ATP production via fatty acid oxidation (FAO). Lipophagy, a selective autophagic process, breaks down LDs and transfers FFAs to mitochondria, aiding cell survival under metabolic stress induced by disrupted oncogenic signaling [128,129]. Moreover, LDs protect against lipid peroxidation, which can trigger ferroptosis [130]. Lipid accumulation in prostate cancer correlates with tumor stage, serving as a diagnostic marker [131]. Hypoxia induces LD formation through HIF-mediated repression of CPT1A, an enzyme crucial for FAO [132]. Besides de novo lipogenesis, increased lipid uptake from the extracellular space also promotes LD accumulation and tumorigenic potential in CSCs [133]. CSCs from various cancers, such as colorectal and ovarian, have higher LD levels than bulk tumor cells. For example, colorectal cancer stem cells show more lipids than normal colon cells and other tumor cell types, with lipid content correlating to CD133 expression and Wnt pathway activation. In this context, CSCs with more LDs demonstrate higher clonogenic and tumorigenic abilities [84]. Similarly, ovarian CSCs (ALDH+/CD133+) have increased LD content compared to ALDH−/CD133− cells. Elevated LDs provide an energy reserve when glycolysis is blocked and protect fatty acids from oxidative damage, supporting CSC survival and proliferation [134]. Conversely, inhibition of phospholipase A2 reduces LDs and induces apoptosis in cancer cells [100].

## 5. Regulation of Amino Acids Metabolism in CSCs

Glutamine, a non-essential amino acid, is crucial for regulating energy balance and metabolic equilibrium during the proliferation of CSCs [135,136,137]. Glutamine regulates redox homeostasis by supporting the synthesis of glutathione (GSH), a key antioxidant in cells, and facilitating NADPH production in many CSC populations. NADPH provides reductive power for the regeneration of ROS-detoxifying enzymes and GSH. Disruption of glutamine metabolism has been shown to inhibit self-renewal, reduce the expression of stemness-associated genes, and decrease pluripotency factors by increasing intracellular ROS levels [138,139,140,141]. The importance of GSH-mediated redox control has been highlighted in certain stem-like cell populations within triple-negative breast cancer. These cells are distinguished by elevated levels of GD2 ganglioside, a novel marker linked to breast cancer stem cells, and they depend on ASCT2 for the import of glutamine [88,101,142]. However, the mechanisms through which glutamine metabolism regulates stem cell properties are not the same across various cancer models. For example, in embryonal carcinoma stem-like cells from both human and mouse sources, a regulatory network involving TAp73, Myc, and SLC1A5 coordinates glutamine uptake and glutathione biosynthesis [86]. Moreover, the synthesis of glutathione driven by glutaminase (GLS) also plays a significant role in the survival and radioresistance of prostate cancer CSCs [143]. Glutamine-derived NADPH is essential for the survival of pancreatic cancer cell lines, such as PANC-1 and SW1990, grown under cancer stem cell conditions. In these cells, glutamine deprivation reduces the expression of stemness-associated genes, impairs self-renewal, and prevents sphere formation [144,145]. Studies have revealed that glutamine depletion can indirectly impact cell signaling pathways through redox imbalance. Liao et al. demonstrated that in A549 non-small cell lung cancer (NSCLC) cells, glutamine starvation reduces the side population (SP) by ROS-induced inhibition of the β-catenin signaling pathway [146]. This results in lower expression of the ABCG2 transporter and the stemness gene SOX2. Additionally, glutamine deprivation increases ROS levels in both SP cells and glioblastoma stem-like cell lines (GSC11, GSC23), impairing their ability to form neurospheres. The increased ROS promotes the degradation of β-catenin and the downregulation of its target genes, Survivin and Axin2. In turn, in some cancer cell lines (epithelial ovarian cancer PA1, OAW42, and colorectal cancer HCT116), glutamine deprivation induces stemness through mitochondrial ROS activation, which triggers the MAPK pathway. This leads to the phosphorylation of dynamin-related protein-1 (DRP1), promoting mitochondrial fragmentation. The fragmented mitochondria increase local ROS, enhancing CSC characteristics, such as CD44 and CD117/CD45 positivity. Interestingly, this process does not involve reduced glutathione synthesis, as no significant changes in cellular GSH are observed [147]. In this scenario, we have to highlight that glutamine serves two main purposes in cancer cells: it helps replenish important TCA cycle intermediates, which are crucial for energy production, and it provides building blocks for making nucleotides essential for cell division [148]. However, in some cancers, like NSCLCs, as we described before, tumor cells may use glucose to replace some of the roles usually played by glutamine [149]. This suggests that in certain environments, cancer cells might be able to adjust their metabolism and use glucose as an alternative to glutamine. In glioblastoma, CSCs rely on both glucose and glutamine. Glucose helps to produce fats, while glutamine maintains the TCA cycle, particularly for generating NADPH, which is crucial for maintaining the cell’s metabolism and survival [150]. Interestingly, CSCs seem to prefer glutamine for their energy needs, especially when dividing rapidly. Because CSCs are so dependent on glutamine for their metabolism, targeting this pathway could be a promising strategy in cancer treatment. By blocking their ability to use glutamine, we could disrupt their energy supply and hinder their growth, making it harder for these cells to survive and proliferate in the tumor [151].

The serine–glycine–one-carbon (SGOC) metabolic network is emerging as a pivotal axis in sustaining the identity and functionality of CSCs [152,153,154,155,156]. This metabolic adaptation supports core stemness features such as self-renewal, high proliferation potential, and epigenetic plasticity. In particular, the de novo serine synthesis pathway (SSP) is frequently upregulated in CSCs across various tumor types, including glioblastoma, breast, and pancreatic cancers [157]. The pathway is initiated by the glycolytic intermediate 3-phosphoglycerate (3-PG), which is diverted away from glycolysis toward serine biosynthesis via the action of phosphoglycerate dehydrogenase (PHGDH) [158]. Subsequent steps involve phosphoserine aminotransferase (PSAT1) and phosphoserine phosphatase (PSPH), culminating in the production of serine. This amino acid serves as a substrate for serine hydroxymethyltransferases (SHMT1/2), which catalyze the reversible conversion of serine to glycine, producing one-carbon units essential for nucleotide biosynthesis and methylation reactions [159,160,161].

In CSCs, these one-carbon units feed into the folate and methionine cycles, enabling the synthesis of purines, thymidylate, and S-adenosylmethionine (SAM). SAM is the universal methyl donor required for the maintenance of the epigenetic landscape characteristic of stem-like tumor cells [162]. Through this link, SGOC metabolism directly influences DNA and histone methylation, allowing CSCs to retain transcriptional programs associated with pluripotency and resistance to differentiation [163]. High levels of PHGDH and SHMT2 have been reported in CSC-enriched subpopulations and correlate with poor prognosis in multiple cancers [164]. Furthermore, oncogenic signaling pathways such as MYC, ATF4, and mTORC1 have been implicated in transcriptionally regulating key SGOC enzymes, reinforcing the pathway’s importance in CSC biology [165]. Therefore, SGOC metabolism provides critical methyl groups necessary for purine ring formation and for the conversion of deoxyuridine monophosphate (dUMP) to deoxythymidine monophosphate (dTMP) by thymidylate synthase [166]. Enzymes such as dihydrofolate reductase (DHFR), thymidylate synthase (TYMS), and amidophosphoribosyltransferase (PPAT) are often upregulated in CSCs, contributing to enhanced nucleotide availability [167,168]. This metabolic adaptation enables CSCs to rapidly replenish nucleotide pools following genotoxic stress and supports their resistance to DNA-damaging agents [169].

## 6. Metabolic Plasticity of CSC Within the TME

The tumor microenvironment (TME) significantly impacts the metabolism of CSCs and LSCs, influencing their metabolic plasticity and response to therapies. Stromal cells, immune cells, the extracellular matrix, and hypoxic conditions within the TME interact with CSCs to support their survival and therapeutic resistance [170]. Stromal cells, such as cancer-associated fibroblasts (CAFs), secrete growth factors and metabolites that provide essential nutrients to CSCs, contributing to their metabolism and resistance to treatments [171]. In the TME, immune cells can modulate the metabolism of CSCs by releasing cytokines and factors that stimulate either glycolysis or OXPHOS, depending on the conditions.

Hypoxia, a common feature of solid tumors, activates HIF-1α signaling, promoting a shift to anaerobic glycolysis and helping cells survive low oxygen levels, thereby enhancing CSC resistance [172,173]. HIF-1α, mTOR, AMPK, and c-Myc are the most studied metabolic key regulators [174]. CSCs exhibit marked metabolic heterogeneity and plasticity, which contributes to their resistance to conventional therapies. CSC subpopulations can dynamically shift between glycolysis, OXPHOS, and lipid metabolism in response to environmental stress, hypoxia, or treatment pressure. For instance, CSCs in hypoxic tumor regions preferentially rely on glycolysis, while those in well-oxygenated areas depend more on OXPHOS to fulfill their energy requirements. This metabolic flexibility is regulated by local signaling cues and pathways such as HIF-1α and mTOR [175].

Under hypoxia, HIF-1α stabilizes and promotes a shift to anaerobic glycolysis, enabling CSCs to produce ATP while minimizing ROS accumulation [63]. mTOR supports CSC growth by driving lipid synthesis, protein translation, and anabolic metabolism [176]. In contrast, AMPK is activated under energy stress and promotes catabolic pathways like autophagy and fatty acid oxidation, aiding CSC adaptation [177,178]. C-Myc enhances glycolysis and nucleotide biosynthesis by upregulating key metabolic enzymes, supporting CSC proliferation [179].

These transcriptional and signaling pathways converge on metabolic effectors that reinforce the stem-like phenotype. For instance, SREBP1 (sterol regulatory element-binding protein 1) regulates genes involved in fatty acid and cholesterol synthesis, supporting membrane biosynthesis and cellular signaling [180,181]. SCD1 catalyzes the formation of monounsaturated fatty acids, critical for membrane fluidity and energy storage, and has been associated with increased CSC survival and tumorigenicity [182,183,184]. CSCs tightly regulate redox balance, maintaining low intracellular ROS levels to preserve stemness, self-renewal, and therapy resistance. This is largely mediated by upregulated antioxidant systems, particularly the glutathione (GSH) and thioredoxin (Trx) pathways [185,186,187]. The GSH system detoxifies ROS via enzymes like glutamate–cysteine ligase and glutathione peroxidase, while the Trx system reduces oxidized proteins and modulates redox signaling [188]. Disrupting these pathways sensitizes CSCs to oxidative stress, reducing viability and tumorigenicity. Notably, dual inhibition of GSH and Trx leads to synergistic cancer cell death, underscoring their therapeutic potential [189]. Furthermore, CSCs exploit metabolic adaptations to support their antioxidant defenses. Enhanced flux through the pentose phosphate pathway (PPP) provides NADPH, a critical reducing equivalent for the regeneration of GSH and Trx [190]. This metabolic reprogramming underscores the interplay between redox regulation and cellular metabolism in CSC maintenance. Targeting the redox balance in CSCs represents a promising therapeutic strategy [191]. Agents that elevate ROS levels or inhibit antioxidant systems can selectively eradicate CSCs while sparing normal stem cells, which possess more robust antioxidant capacities [135]. This metabolic plasticity is a key factor in treatment resistance and disease relapse, as CSCs can transition to an alternative metabolism to escape therapeutic effects, becoming dormant or quiescent for extended periods [192].

## 7. Epigenetic Regulation of Metabolic Pathways in CSCs

It is now well established that oncogenic mutations driving tumor initiation and progression are closely associated with widespread epigenetic disruptions. These include global DNA hypomethylation, localized hypermethylation at CpG (cytosine–phosphate–guanine) island promoters, altered histone modification profiles, and changes in nucleosome positioning [193]. Epigenetic regulation encompasses reversible chemical modifications to DNA and histone proteins that modulate gene expression without altering the nucleotide sequence. Principal mechanisms include DNA methylation, post-translational histone modifications—such as acetylation, methylation, phosphorylation, and ubiquitination—and the action of non-coding RNAs. These processes influence chromatin architecture and accessibility, thereby regulating transcriptional activity [194,195]. Epigenetic states are shaped by both genetic predisposition and environmental exposures, linking them to various pathological conditions, notably cancer [193]. Epimutations—aberrant epigenetic changes—can impair gene regulation, promoting genomic instability and cancer progression [196,197,198,199]. They may drive oncogenesis by evading growth control, resisting apoptosis, sustaining proliferation, and enhancing metastasis [137,141].

DNA methylation, catalyzed by DNA methyltransferase (DNMT) enzymes, mainly targets cytosine residues within CpG islands, typically located in gene promoter regions. In cancer, global DNA hypomethylation promotes chromosomal instability and oncogene activation, while focal hypermethylation silences tumor suppressor genes [200,201]. 5-hydroxymethylcytosine is generated through the oxidation of 5-methylcytosine and plays a role in maintaining stem cell plasticity and contributing to tumor progression [202].

Acetylation of histone H3 at lysine 9 (H3K9ac) promotes gene activation, whereas trimethylation at H3K27 (H3K27me3) is linked to gene silencing [203]. Dysregulation of these marks contributes to tumor development by altering chromatin dynamics [204,205]. Enzymes that modify histones are, therefore, promising targets for therapy, and histone deacetylase inhibitors (HDACis) are already used clinically in cancer treatment.

Non-coding RNAs (ncRNAs) are important epigenetic modulators that influence gene expression without altering DNA sequences [206]. Long ncRNAs (typically over 200 nucleotides in length) regulate transcription by interacting with chromatin-modifying complexes at specific genomic loci [147]. In contrast, microRNAs (miRNAs -17–25 nucleotides) act mainly at the post-transcriptional level by binding to untranslated regions of target mRNAs, leading to their degradation or translational repression through RNA interference pathways [207]. Both lncRNAs and miRNAs contribute to the precise regulation of gene networks involved in tumor initiation and progression [208,209,210,211].

Epigenetic alterations are increasingly recognized as key regulators of CSC identity. A well-characterized example is the epithelial-to-mesenchymal transition (EMT), which has been associated with the acquisition of CSC-like properties. In breast cancer, EMT correlates with elevated expression of CSC markers (e.g., CD44high/CD24low), enhanced self-renewal capacity, and increased tumor-initiating potential [212,213,214]. Emerging evidence indicates that EMT is governed, in part, by epigenetic mechanisms—particularly through modifications that regulate the transcription of mesenchymal factors such as ZEB1—highlighting the dynamic interplay between epigenetic control and CSC plasticity [215].

## 8. Optimizing Strategies for CSC Targeting

Targeting CSCs holds promise for long-term cancer remission. Traditional identification methods rely on flow cytometry and functional assays, but surface and cytoplasmic markers often lack specificity due to technical limitations and tumor heterogeneity. Incorporating metabolic markers may enhance the accuracy of CSC detection by capturing their distinct functional properties [216,217]. To evaluate metabolic therapies, experimental models are essential. While in vitro systems—such as CSC-enriched cultures and cell lines—offer initial insights, they often fail to replicate the tumor microenvironment. In contrast, patient-derived xenografts (PDX) and organoids provide more physiologically relevant models for assessing therapeutic responses [218].

Enhanced mitochondrial biogenesis can be a marker for identifying cancer cells with enhanced self-renewal capacity across various cancers [219,220]. Specifically, using fluorescent probes like MitoTracker allows for effective identification of CSCs regardless of their metabolic phenotype, whether glycolytic or reliant on OXPHOS. For instance, metabolic profiling of MCF7 breast cancer cells using MitoTracker has shown that a high mitochondrial mass is associated with CSC populations that have anabolic properties [72]. Mitochondrial enrichment in breast cancer stem cells enhances DNA repair and may help them resist anticancer treatments [221]. AML cells increase their mitochondrial mass by transferring mitochondria from bone marrow stromal cells, which supports survival and long-term culture potential [222]. Mitochondrial movement and mitophagy contribute to cancer stemness, and measuring mitochondrial membrane potential helps identify CSCs [223]. In breast cancer, a molecular toolkit has been developed for CSC identification based on markers like increased mitochondrial biogenesis, ROS production, and NADH levels [224].

Conventional therapies often fail to eradicate CSCs as they primarily target differentiated cancer cells. Therefore, developing strategies to specifically eliminate CSCs is a key challenge in modern cancer research (Table 1). A crucial step in this process is distinguishing CSCs from normal stem cells, as this differentiation could lead to the development of targeted treatments that spare healthy cells. Understanding the fundamental metabolic traits could lead to the identification of potential therapeutic targets [155,156]. Inhibitors of mTOR, for instance, impair lipid and protein biosynthesis, while agents like metformin and 2-deoxy-D-glucose (2-DG) disrupt glycolysis and mitochondrial metabolism [225]. The combination of 2-DG with fenformin, an AMPK activator, has shown enhanced antitumor efficacy in colon cancer models, including patient-derived xenografts. Metformin, an anti-diabetic agent, has been shown to suppress glycolysis, the TCA cycle, and nucleotide synthesis, leading to reduced CSC formation and tumor growth [226]. In a Phase II clinical trial (NCT01579812), metformin led to a 2.4-fold reduction in ovarian ALDH+CD133+ CSCs, increased cisplatin sensitivity, and improved both progression-free and overall survival [227]. Despite these promising advances, CSCs can develop resistance to metabolic therapies. Consequently, current research focuses on combinatorial approaches that simultaneously target multiple metabolic pathways or integrate metabolic inhibitors with immunotherapy, aiming to overcome compensatory survival mechanisms [228]. In pancreatic cancer, metformin induces cell death by inhibiting mitochondrial complex I, preventing the metabolic shift to glycolysis [229,230]. However, their survival is compromised when this metabolic adaptability is disrupted, as observed in K-ras-resistant pancreatic CSCs. Accumulating evidence supports the inclusion of metabolic alterations as a hallmark of cancer, positioning the metabolic profile of CSCs as a crucial target for therapeutic intervention [231]. High-throughput analysis and systems biology have accelerated the discovery of key metabolic regulators essential for CSC maintenance. Altogether, the distinctive metabolic characteristics of CSCs hold potential for therapeutic intervention without compromising normal tissue homeostasis.

## 9. Conclusions

The CSC model suggests that a small, metabolically adaptable subpopulation drives tumor growth and therapy resistance. CSCs can shift between glycolysis and mitochondrial metabolism, enabling survival under stress and in a quiescent state.

Targeting CSC metabolism is a promising strategy, but their adaptability and dormancy complicate eradication. A comprehensive strategy that targets multiple metabolic pathways is essential to effectively eliminate CSCs. Further research is essential to identify specific CSC markers, distinguish their metabolism from normal stem cells, and develop effective therapies.

## Figures and Tables

**Table 1:** Putative metabolic strategies to target CSCs. OXPHOS: oxidative phosphorylation; FASN: Fatty Acid Synthase; CPT1: Carnitine Palmitoyltransferase 1; ROS: reactive species of oxygen; mTOR: mammalian target of rapamycin.

Therapeutic Strategy	Drug	Clinical Trials
Glycolysis	2-DG Lonidamine	NCT00633087 NCT02758860
OXPHOS	Metformin Oligomycin Complex I inhibitors	NCT01579812 none NCT02882321, NCT03291938
Glutaminolysis	CB-839 (glutaminase inhibitor)	NCT02071862, NCT03163667, NCT03872427
Lipogenesis	Orlistat (FASN inhibitor)	none
Fatty acid β-oxidation	Etomoxir (CPT1 inhibitor)	none
ROS production	Salinomycin Fenretinide	none NCT00646230
mTOR	Sirolimus	NCT03433183
	Everolimus	NCT00876395, NCT01007942,
		NCT04485559
	Temsirolimus	NCT01614197, NCT03433183
	Ridaforolimus	NCT00538239

## Data Availability

No new data were created or analyzed in this study. Data sharing is not applicable to this article.
